# Remote sensing inversion on heavy metal content in salinized soil of Yellow River Delta based on Random Forest Regression—a case study of Gudao Town

**DOI:** 10.1038/s41598-024-62087-y

**Published:** 2024-05-16

**Authors:** Pingjie Fu, Xiaotong Li, Jiawei Zhang, Chijie Ma, Yuqiang Wang, Fei Meng

**Affiliations:** 1 School of Surveying and Geo-Informatics, Shandong Jianzhu University, Jinan, 250101 China; 2 College of Geodesy and Geomatics, Shandong University of Science and Technology, Qingdao, 266590 China; 3 Disaster Reduction Center of Shandong Province, Jinan, 250101 China

**Keywords:** Environmental sciences, Environmental social sciences

## Abstract

To explore the potential of using the mineral alteration information extracted by remote sensing technology to indirectly estimate the heavy metal content of salinized soil, 23 sampling points were uniformly set up in the town of Gudao in the Yellow River Delta as the research area in 2022. The concentrations of seven heavy metals, Cr, Cu, Pb, Zn, As, Mn and Ni, at the sampling points were determined in laboratory tests. Spectral derivative indices, topographic factors, and mineral alteration information (iron staining, hydroxyl, and carbonate ions) were extracted and screened as modeling factors using Sentinel 2 imagery. An inverse model of heavy metal content was constructed using the random forest algorithm, and the model accuracy was evaluated using the cross-validation method. The results of the study show that: (1) Hydroxyl and carbonate ion alteration can be effectively used for the inversion of soil As and Ni content in this study area. Iron-stained alteration can be used as a modeling factor in the inversion of Cr, Cu, Pb, Zn, and Mn concentrations. (2) The inclusion of alteration information improves the accuracy of heavy metal content inversion. The Cu concentration was verified to be the best predictor, with an RMSE of 3.309, MAPE of 11.072%, and R^2^ of 0.904, followed by As, Ni, and Zn; the predictive value of Mn, Cr and Pb was average. (3) Based on the results of concentration inversion, the high concentration areas of As, Ni, and Mn are primarily distributed on both sides of the river and around lakes and ponds. The high-concentration areas of Zn were mainly distributed in the farmland areas on both sides of the river. Areas with high concentrations of Cu were mainly distributed in the eastern oil extraction area, both sides of the rivers, and around lakes.

## Introduction

Soil salinization has a serious impact on the ecological environment of the Yellow River Delta wetlands. In recent years, the sediment entering the Yellow River has decreased sharply, and the rate of seawater erosion has changed, resulting in the shrinkage of the wetlands due to seawater backwater, which has aggravated the salinization of the delta soils. To some extent, it constrains the development of local agriculture. Simultaneously, deepening salinization leads to increased soil vulnerability. Human activities such as oil production and mining have resulted in some heavy metals entering coastal beaches. According to the data from the 2019 ecological geochemical survey of the lower Yellow River Basin in Shandong Province, the contents of the heavy metals As, Cr and Ni in the soils within Dongying City exceeded the surface soil elemental background values for this heavy metal element in Shandong Province. Although As is not a heavy metal, it is usually included in the category of heavy metals for discussion because its behavior and source as well as its harm are similar to those of heavy metals. According to previous studies, most of them used the concentration of heavy metal elements such as Cu, Pb, Zn, Cr, Ni, Mn, Cd, and Hg as important indicators of soil quality^1,2^. Therefore, Cu, Pb, Zn, Cr, Ni, Mn, and As were selected as inversion objects in this paper to provide technical support for the in-depth study of the spatial distribution and large-scale monitoring of heavy metal concentrations in salinized soils in the Yellow River Delta. It can provide a scientific basis for ecological environmental protection, land quality management, rational use of resources, and agricultural production in the region.

In recent years, researchers have studied a variety of methods for monitoring soil heavy metal content in typical areas. Examples include direct assessment using the sample analysis method^3^, X-ray fluorescence (XRF) in situ detection^4^, pollution evaluation and source analysis^5^, spatial distribution of GIS methodologies, and inversion of heavy metal content in multi-source remote sensing images^6,7^. As an advanced Earth observation technology, remote sensing has the advantages of objectivity and reality, long time series, large area, low cost, simplicity high efficiency. Combined with the rapid development of computer technology, this technology, and plays an important role in the assessment of heavy metal content in soil. Hyperspectral remote sensing data have a high spectral resolution. The accuracy of soil heavy metal quantitative inversion can be improved by optimizing feature extraction^8^, band combination^9^, and training models^10^. Among these, spectral data obtained using portable ground object spectrometers have high accuracy, which is convenient for model construction and mechanism analysis. However, achieving model transposition for large-scale images is difficult. Satellite-based hyperspectral data facilitate large-area monitoring but often require complex data preprocessing because of limitations in spatial resolution and signal-to-noise ratio. Guo et al. used the DS algorithm to correct the Zhuhai-1 hyperspectral image to match the laboratory spectrum. Then the Cr in soil was predicted based on the combined SNV + UVE + SVR model, and the inversion was effective^11^. Sun et al. inverted the Zn content by extracting the characteristic bands of organic matter and clay minerals that adsorb the soil heavy metal Zn. The results showed that VNIR hyperspectral data were superior to VNIR-SWIR hyperspectral data in predicting and localizing Zn concentrations in soil^12^. Airborne hyperspectral data solve the problems of spatial resolution and data accuracy, enabling high-precision inversion of soil heavy metal contents at a small scale. However, the cost of data acquisition is high, and the maintenance cycle of the instrument is long. Multispectral remote sensing imagery is advantageous in terms of spatial resolution, processing efficiency, time series of available data, and cost. Current research mainly focused on constructing an inversion model of soil heavy metal content by extracting and screening the characteristic bands, spectral derivative indices^7,13,14^, and topographic factors (DEM, slope, and aspect) of heavy metal concentrations. Yang et al. proposed a network model based on transfer learning theory and a back propagation (BP) network optimized by genetic algorithm (GA). The visible and near-infrared spectral data from Landsat8 satellite images, and modeling factors selected by the digital elevation model were used to predict Cu and Pb contents. The results show that the proposed Tr-GA-BP network performs well^15^. Wang et al. proposed an optimization of the commonly used partial least squares regression (PLS) method to invert the Ni, Cu, and Zn contents of tailings ponds and their surroundings by using a variety of remote sensing indices and the modeled heavy metals as modeling factors based on GF-2 image. Compared to the traditional PLS, the goodness-of-fit of Ni, Cu and Zn was improved by 0.0852, 0.2291 and 0.2919, respectively^16^. Iron oxides and clay minerals have a certain adsorption effect on soil heavy metals^17^. Iron-stained, hydroxyl (–OH), and carbonate ion (CO_3_^2−^) alteration information can reflect the distribution of the two types of minerals, and the technology of alteration mineral information extraction based on remotely sensed images is very mature^18,19^. However, the alteration of mineral information has not yet been applied to the image inversion of soil heavy metal content. Yang et al. extracted mineralization and alteration information in the Xinjiang Tashkurgan Prefecture area using mineral indicators and mask models based on ASTER images. Statistical analysis with existing geological data identified three mineralized prospecting targets^20^. Zhang et al. used the GF-5 visual infrared spectroscopic imager (VIMS) to extract iron staining and hydroxyl alteration. It was found the alteration information extracted from VIMS image is consistent with the alteration extracted from TM image, and it is more accurate^21^. Sentinel-2A satellite data has more advantages in spatial resolution and spectral resolution than Landsat series images. Li et al. used Sentinel-2A satellite data to analyze the spectral combination responses of different types of alteration, and then selected the optimal principal component analysis scheme to extract alteration information^22^. According to previous studies, the extraction of alteration information is dominated by methods such as band ratio, principal component analysis, and independent component analysis (ICA)^23,24^. In particular, the principal component analysis method has the advantages of simple realization, fast speed, good effectiveness, and robustness, which make a significant contribution to the extraction of alteration information^25^.

To investigate the potential of alteration information in the inversion of heavy metal content in salinized soils, this study used the town of Gudao in the Yellow River Delta as the research area to obtain seven heavy metal concentrations of soil Cr, Cu, Pb, Zn, As, Mn, and Ni from 23 sampling points. Sentinel-2A satellite imagery and spectral derivative indices, topographic factors, and alteration information to extract and screen modeling factors. A random forest regression model was constructed to predict soil heavy metal content, and the accuracy of the estimation results was evaluated. To investigate the effectiveness of mineral alteration information extracted from multispectral remote sensing images in soil heavy metal inversion, with a view to providing new ideas and methods for rapid and efficient large-area soil quality monitoring, and to improve the technical level of multispectral remote sensing in support of soil environmental quality research.

## Overview of the study area and data acquisition

### Overview of the study area

**Figure 1:**
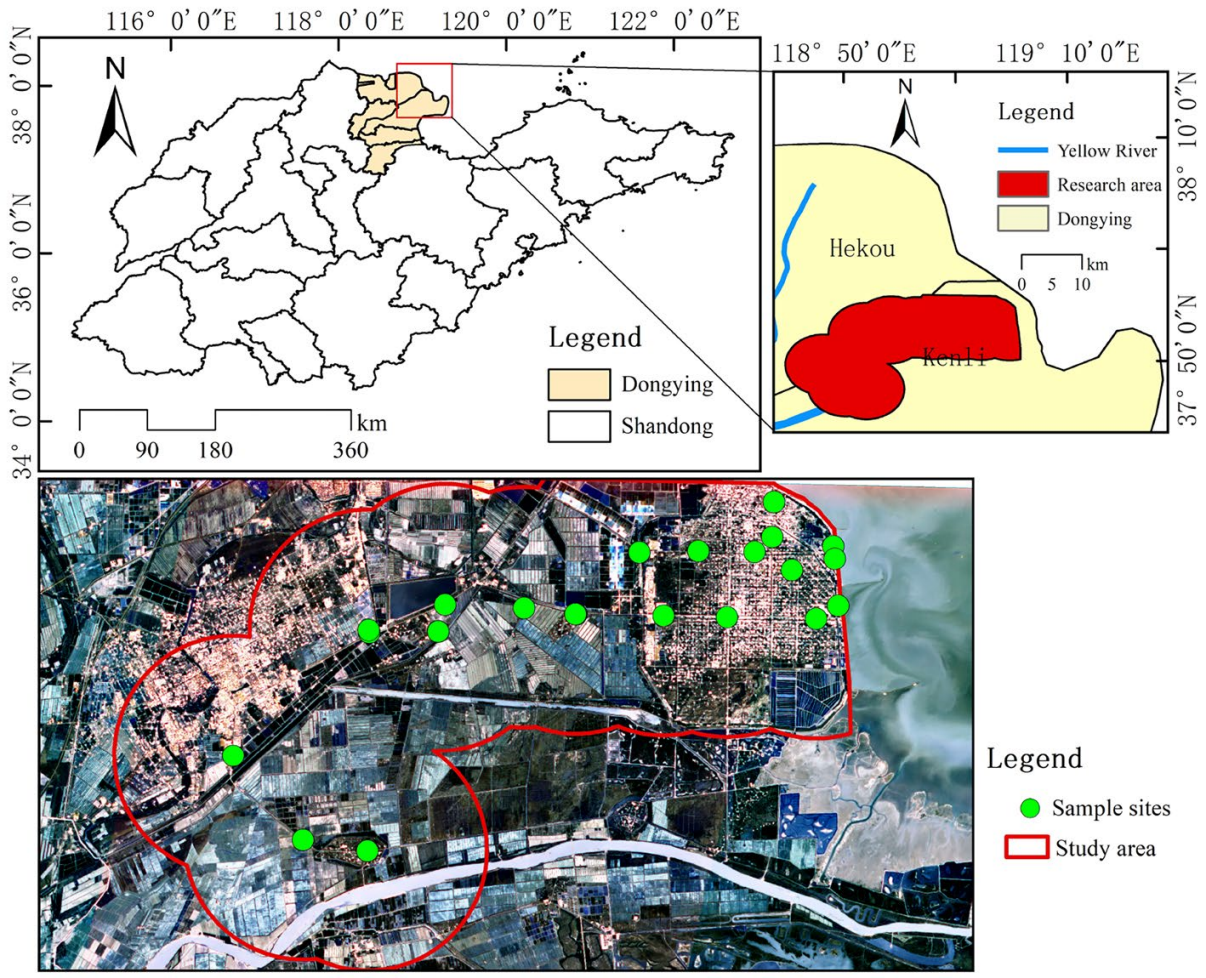
Geographic location of the research area.

The study area was the town of Gudao, northeast of Dongying City, Shandong Province, China, located in the Yellow River Delta. Surrounded by the Bohai Sea in the north and the Laizhou Bay in the east, with the geographical coordinates of 118° 39′–119° 8′ E and 37° 47′–37° 84′ N, it has a total area of 159.46 square kilometers and is separated from the Jiaodong Peninsula by the sea (Fig. 1). The town is located in the semi-arid warm-temperate East Asian monsoon zone, through the statistics of meteorological data from 2001 to 2020, in which the average annual temperature is 12.1 °C, 12.9 °C is the highest average annual temperature and 10.9 °C is the lowest average annual temperature, an annual precipitation of 347–813.2 mm, and an annual sunshine of 2600–2800 h. Underground oil resources are rich, and the soil above the ground is fertile and well-irrigated, making it suitable for growing crops such as rice, wheat, corn, and soybeans. The traffic is well-developed, and the internal roads are vertically and horizontally aligned.

### Data acquisition and preprocessing

1. Soil sample data

Soil samples were collected in the field on July 25–27, 2022 in Gudao, and were mainly distributed in the eastern part of the study area in the oil extraction area, in the central part of the agricultural area, and to a lesser extent, on the sides of riverbanks and pits, at 23 sampling sites. Surface weeds and stones were removed by digging to a depth of 0–30 cm using a shovel. Soil samples were collected in polyethylene plastic bags weighing approximately 2.5 kg per sampling point. Using a Global Navigation Satellite System (GNSS) receiver to receive signals from a continuously operating reference station (CORS), and using Real-Time Kinematic (RTK) method to locate the sampling point, the coordinate system is WGS-84. Sealed bags are labeled with the time of sampling, latitude and longitude, and sample number. Transportation in black plastic bags protected from light at the end of sampling. Afterwards, the collected samples are sent back to a professional testing organization for testing. First, the samples were removed from impurities such as stones and plant fragments. Then, soil samples were dried after natural air drying, grinded with a glass rod and passed through a 100 mesh nylon aperture sieve, and placed in clean sample bags. A German-made wavelength dispersive X-ray fluorescence spectrometer (Bruker S8 TIGER) was utilized for the detection of heavy metal elements in soil samples. The metal element concentrations were detected using wavelength-dispersive X-ray fluorescence spectrometry (HJ780-2015). The detection limits were 3.0 mg/kg for Cr, 10.0 mg/kg for Mn, 1.5 mg/kg for Ni, 1.2 mg/kg for Cu, and 2.0 mg/kg for Zn, As, and Pb.

**Figure 2:**
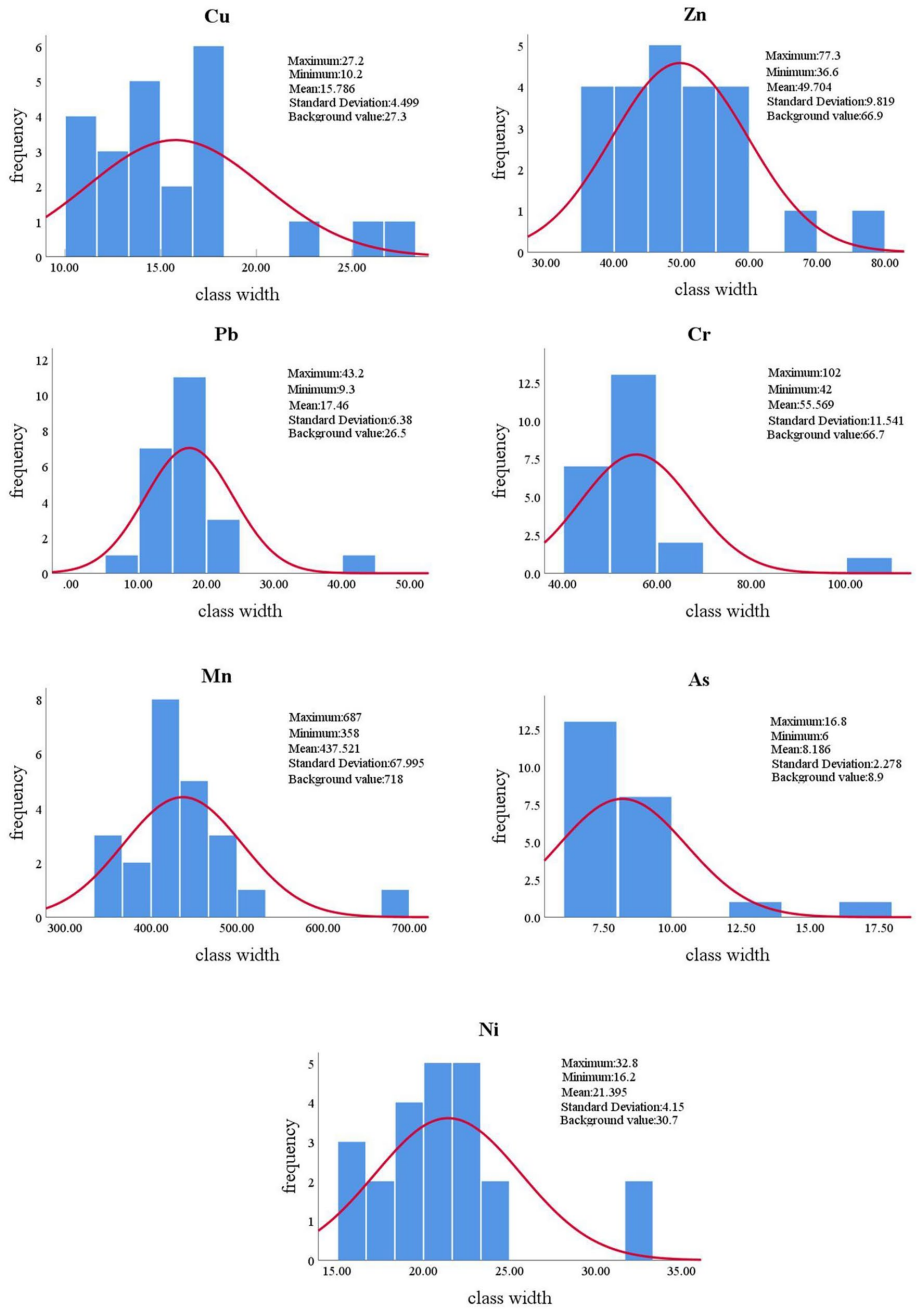
Elemental content histograms.

The numerical characteristics of the seven metal elements were determined based on the results of the laboratory tests. The extreme values, arithmetic means, and standard deviations of the metal contents were selected as statistical indicators and compared with the background values of soil chemistry in Dongying City. The calculated comparisons showed that the percentages of samples with Cu, Pb, Zn, Cr, Mn, As, and Ni contents exceeding the soil chemical background values in Dongying were 0%, 10%, 2.5%, 5%, 0%, 7.5%, and 10%, respectively. Based on the distribution of metallic elements in the study area, a histogram rectangle with group distance as the bottom edge and frequency as the height was plotted to show the content of each metallic element at the 23 sampling points and the exact distribution of the elements (Fig. 2).

2. Remote sensing image data

The Sentinel-2A satellite was launched on June 23, 2015, using a Vega launch vehicle carrying a multispectral imager. It is a high-resolution multispectral imaging satellite with an altitude of 786 km, coverage of 13 spectral bands, three types of spatial resolution (10, 20, and 60 m) and a width of 290 km. Sentinel-2A imagery combines the strengths of the Landsat and Spot satellites with high-resolution multispectral features and relatively stable quality. Therefore, in this study, Sentinel-2A data on October 23, 2022 were selected for an inversion study of soil heavy metal content in the Yellow River Delta region. Images covering the entire study area were downloaded from the official website of the European Space Agency. The downloaded Sentinel-2A remote sensing images were imported into SNAP and exported in ENVI format after 10 m resampling. Band fusion is performed on the exported files in the ENVI platform to obtain remote sensing images displayed in real bands. As the Sentinel-2A remote sensing imagery acquired is at the L2A level and have been processed for radiometric calibration and atmospheric correction, and only cropping was required before image processing and analysis. Water disturbance information was extracted by MNDWI, and band3 and band11 were selected to participate in the calculation. A segmentation threshold of − 0.12 < WNDWI < − 1 was used to create a water mask file, and the image was masked using the mask file.

## Theory and methodology

### Modeling factors

1. Spectral derivative indices

Various vegetation covered on the ground absorbs some heavy metal elements from the soil during its growth process, as well as soil moisture, brightness, and color, can affect satellite sensors to a certain extent when they receive reflected waves from the ground. Currently, the use of vegetation indices to invert land-cover change is gradually becoming an important research tool for understanding global environmental change^26,27^. Therefore, in this study, a variety of spectral indices reflecting the surface conditions, also known as spectral derivative indices, were selected to enhance specific information in remote sensing images.

The spectral derivative indices selected for the study were the Normalized Difference Vegetation Index (NDVI), Clay Mineral Ratio (CMR), Modified Normalized Difference Water Index (MNDWI), Ratio Vegetation Index (RVI), Difference Vegetation Index (DVI), modified soil-adjusted vegetation index (MSAVI), Enhanced Vegetation Index (EVI), and Red-Green Ratio Index (RGRI), as shown in Table 1.

**Table 1:** Spectral index calculation formulas.

Spectral index	Calculation formula	References
NDVI	(NIR − Red)/(NIR + Red)	^28^
CMR	SWIR1/SWIR2	^29^
MNDWI	(Green – SWIR1)/(Green + SWIR1)	^30^
RVI	NIR/Red	^31^
DVI	NIR − Red	^37^
MSAVI	$\left[\left(2{\text{NIR}}+1\right)-\sqrt{({2{\text{NIR}}+1)}^{2}-8({\text{NIR}}-{\text{Red}})}\right]/2$]	^38^
EVI	2.5[(NIR − Red)/(NIR + 6Red − 7.5Blue + 1)]	^39^
RGRI	Red/Green	^32^

*Blue* blue band reflectance, *Green* green band reflectance, *Red* red band reflectance, *NIR* near-infrared band reflectance, *SWIR1* short-wave infrared 1 reflectance, *SWIR2* short-wave infrared 2 reflectance.

2. Topographic factors

The ASTER GDEM Digital Elevation Model (DEM), which covers the entire study area at a resolution of 30 m, was downloaded from the Geospatial Data Cloud website. The ASTER GDEM is an easily accessible, high-precision DEM that covers virtually all of the Earth's landmass and is available to all users, regardless of the size or location of their target area. The DEM data were further used for terrain factor extraction, including aspect and slope.

3. Alteration information

The alteration of surrounding rocks refers to the process by which rocks and minerals undergo changes to their original physical and chemical properties due to hydrothermal activity, leading to the formation of new mineral assemblages^33^. Iron oxides and clay minerals have a certain accompanying relationship with heavy metals in soil, and information on iron-stained alteration, –OH, and CO_3_^2−^ alteration may reflect the distribution of the two types of minerals. Principal component analysis (PCA) is one of the most commonly used methods for the extraction of alteration information. This is the process of reducing the correlation between images using a linear transformation to represent the information in each band with the fewest comprehensive indicators, thus highlighting the useful information contained in the image. By performing PCA on the water masked image, the information is mainly concentrated in the first component so that the magnitude of the contribution of the generated principal components can be reflected in the magnitude of the eigenvector matrix weights^34,35^. After specifically analyzing the actual situation of the study area, we chose to extract iron stained, –OH, and CO_3_^2−^ alteration information.

In 1978, Hunt et al. found that minerals containing ions or ionophores such as Fe^2+^, Fe^3+^, –OH, and CO_3_^2−^ have unique absorption properties in the visible-near-red band by examining the reflectance spectral characteristics of minerals^36^. With the help of rock and mineral spectral curves obtained from the U.S. Geological Survey (USGS) standard spectral library, the characteristic absorption peaks of the iron-stained minerals occur at 0.40–0.50 μm, 0.80–0.92 μm, 1.39–1.41 μm, and 1.90–1.92 μm, and the characteristic reflectance peaks are mainly distributed at 0.65–0.72 μm, 1.23–1.30 μm, and 1.60–1.70 μm. The characteristic absorption bands of –OH and CO_3_^2−^ ionophores were generally distributed at 0.86–0.98 μm, 1.32–1.44 μm, and 2.15–2.55 μm, in which the most significant absorption feature was located at the vicinity of 2.33 μm, and the characteristic reflectance bands were mainly distributed at 0.49–0.58 μm, 1.60–1.70 μm, and 2.00–2.14 μm.

Based on the spectral characteristics of iron-stained minerals, after multiple band selections, two absorption bands at 0.40–0.50 μm and 0.80–0.92 μm, as well as two reflection bands at 0.65–0.72 μm and 1.60–1.70 μm were selected. These correspond to bands 2, 8, 4, and 11 of Sentinel-2A imagery, respectively. PCA (Table 2) was performed to extract information related to the alterations in iron staining. Based on the characteristics of the eigenvectors of the anomalous principal components, the absolute values of the loading coefficients for bands 2 and 4 must be relatively large and have opposite signs: one negative and one positive. Additionally, the loading coefficients for bands 8 and 11 should have opposite signs: one negative and one positive. By analyzing the PCA eigenvector matrix of the Sentinel-2A images in the study area, the loading coefficients of bands 2, 8, 4, and 11 in the principal component of PC4 were found to be consistent with the requirements. Thus, we conclude that the iron-stained alteration information was located in the PC4 component.

**Table 2:** PCA eigenvectors for Sentinel-2A image: iron-stained alteration.

Alteration information	Feature vectors	Band 2	Band 4	Band 8	Band 11
Iron-stained alteration	PC1	0.287706	0.412901	0.613045	0.609027
	PC2	0.554523	0.599958	− 0.568562	− 0.222855
	PC3	− 0.126458	− 0.269277	− 0.54087	0.786739
	PC4	− 0.770544	0.630122	− 0.091502	0.02891

Based on the spectral characteristics of –OH and CO_3_^2−^ minerals, two absorption bands of 0.86–0.98 μm and 2.15–2.55 μm, and two reflection bands of 0.49–0.58 μm and 1.60–1.70 μm, corresponding to bands 8, 12, 2, and 11 of the Sentinel-2A image, were finally selected by several band selections and were subjected to PCA (Table 3) to extract the –OH and CO_3_^2−^ alteration information. Depending on the characteristics of the eigenvectors of the anomalous principal components, the signs of the loading coefficients of band 2 and band 8 should be opposite: one positive and one negative, and the signs of the loading coefficients of band 11 and band 12 should also be oppositive: one positive and one negative. The results of the analysis presented in Table 3 show that the loading coefficients of bands 8, 12, 2, and 11 in the PC4 principal component of the Sentinel-2A image in the study area meet the requirements. Therefore, the –OH and CO_3_^2−^ alteration information of the Sentinel-2A image of Gudao Town was also determined to be located in the PC4 component.

**Table 3:** PCA eigenvectors for Sentinel-2 image: –OH and CO_3_^2−^

Alteration information	Feature vectors	Band 2	Band 8	Band 11	Band 12
–OH and CO_3_^2−^	PC1	0.277139	0.590494	0.590494	0.475214
	PC2	0.203867	− 0.778902	0.250637	0.537524
	PC3	0.855006	0.082575	− 0.510898	0.0336
	PC4	0.388069	− 0.194447	0.57226	− 0.695781

4. Complex covariance test

A strong correlation between the factors distorts the model estimation. Therefore, the above factors must be tested for complex covariance to prevent the existence of serious complex covariance among them.

The Variance inflation test (VIF) was used to test the linear correlation between the factors. The formula is as follows:

1 $$ VIF_{j} = \frac{1}{{1 - R_{j}^{2} }} $$

The test statistic used to examine the explanatory ability of the model is R^2^ (sample decidability coefficient). The magnitude of R^2^ determines the degree of correlation between factors. A smaller R^2^ value indicates that this factor is less linearly correlated with the other factors. If the VIF value is greater than 5, it indicates the presence of multicollinearity; if the VIF value is greater than 10, it indicates the presence of severe collinearity and the factor needs to be discarded.

### Random Forest Regression model (RFR model)

Random Forest Regression (RFR) is the combination of multiple CART decision trees as weak learners to form a "forest" to make predictions on a dataset^40^. A single decision tree can only take a single classifier when making decisions, which inevitably results in disadvantages, such as the decision tree being able to select only one type of attribute to analyze at a time or overfitting the model to accommodate noise. To address these shortcomings, the algorithm builds a forest using randomization, in which the decision trees in the forest are not associated with each other. Multiple decision trees constructed to average errors and improve prediction accuracy are the core concepts of random forests.

To determine the accuracy of the model, three accuracy indicators used in this study were root mean square error (RMSE), mean absolute percentage error (MAPE), and R^2^.

RMSE is the square root of the ratio of the square of the deviation of the predicted value from the true value to the number of observations, which is of the same order of magnitude as the true value; it measures the deviation of the predicted value from the true value and is more sensitive to outliers in the data. The formula is as follows:

2 $$ RMSE = \sqrt {\frac{1}{n}\sum\limits_{i = 1}^{n} {\left( {\widehat{y}_{i} - y_{i} } \right)^{2} } } $$

MAPE is the average of the deviation of the predicted value from the true value and the absolute value of the true value, and is a relative metric that is commonly used as a statistical measure of prediction accuracy. The formula is as follows:

3 $$ MAPE = \frac{100\% }{n}\sum\limits_{i = 1}^{n} {\left| {\frac{{\widehat{y}_{i} - y_{i} }}{{y_{i} }}} \right|} $$

The R^2^ statistic is an important measure of the goodness-of-fit of the model and is calculated as the ratio of the regression sum of squares to the total sum of squares. The numerical values reflect the relative contributions of the regression. The formula is as follows:

4 $$ R^{2} = \frac{{\sum\nolimits_{i = 1}^{n} {(\widehat{{y_{i} }} - \overline{{y_{i} }} )^{2} } }}{{\sum\nolimits_{i = 1}^{n} {(y_{i} - \overline{{y_{i} }} )^{2} } }} $$

The range for RMSE and MAPE values is [0, + ∞); when the gap between the predicted and true values is smaller, the closer the value is to 0, the higher the model accuracy; therefore, the smaller the RMSE and MAPE values of the model, the higher the model efficacy. Compared to RMSE, MAPE provides a more realistic reflection of the deviation between the predicted and actual values, presented as a percentage to enhance user understanding. However, the calculation is limited when the actual values are close to or equal to zero. By contrast, RMSE is applicable to any dataset and is more sensitive to outliers in the data. The R^2^ value ranges from 0 to 1, with values closer to 1 indicating a higher model accuracy and stronger predictive capability.

## Results and analysis

### Modeling factor extraction and screening

**Figure 3:**
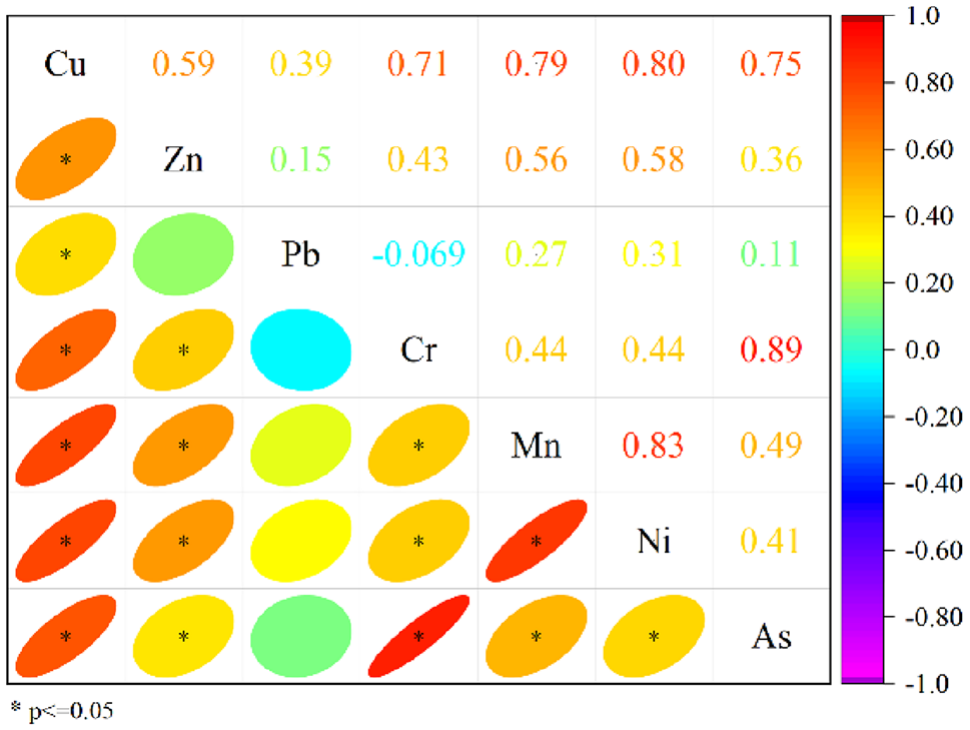
Correlations between heavy metal content.

The correlations between the contents of the seven heavy metals were analyzed to obtain a comparable matrix of correlation coefficients, indicating a correlation between the variables (Fig. 3). Figure 3 shows that the correlations of As, Mn, Ni, Cu, and Cr were better.

Spectral data, including six spectral bands and eight derived spectral indices, were extracted from the Sentinel-2A image of Gudao and analyzed for correlations between the concentration of each heavy metal and the spectral data (Table 4). As shown in Table 5, the correlation between the elemental concentrations of Mn, Ni, As, Cu, and Zn and the six spectral bands was better, whereas the correlation between Pb and Cr and the six spectral bands was worse. Regarding spectral indices, the elemental concentration of Mn correlated well with MSAVI, MNDWI, RGRI, and NDVI, and the concentration of Ni correlated well only with MNDWI; all eight spectral indices correlated well with As, and the elemental concentrations of Cu and Zn correlated well with MSAVI, MNDWI, and RGRI.

**Table 4:** Correlation coefficients between heavy metal content and spectral data.

	Mn	Ni	As	Cu	Zn	Pb	Cr
Blue	− 0.446*	− 0.398	− 0.635**	− 0.519*	− 0.550**	0.044	− 0.107
Green	− 0.509*	− 0.455*	− 0.649**	− 0.572**	− 0.628**	− 0.003	0.212
Red	− 0.490*	− 0.419*	− 0.604**	− 0.530**	− 0.598**	0.008	0.209
NIR	− 0.408	− 0.317	− 0.568**	− 0.452*	− 0.478*	− 0.015	0.151
SWIR1	− 0.432*	− 0.366	− 0.575**	− 0.516*	− 0.511*	− 0.050	0.198
SWIR2	− 0.456*	− 0.408	− 0.606**	− 0.547**	− 0.558**	− 0.054	0.245
CRM	− 0.158	− 0.037	− 0.296	− 0.184	− 0.137	0.012	− 0.111
EVI	− 0.158	− 0.037	− 0.296	− 0.184	− 0.137	0.012	− 0.111
DVI	− 0.092	0.041	− 0.374	− 0.149	− 0.018	− 0.111	− 0.024
MNDWI	− 0.289	− 0.222	− 0.536**	− 0.403	− 0.360	− 0.054	0.033
MSAVI	− 0.314	− 0.125	− 0.369	− 0.230	− 0.207	− 0.111	− 0.139
NDVI	0.234	0.060	0.329	0.173	0.132	0.088	0.123
RGRI	− 0.269	− 0.180	− 0.543**	− 0.343	− 0.397	0.078	0.075
RVI	− 0.155	− 0.001	− 0.278	− 0.117	− 0.056	− 0.062	− 0.097

*Significant correlation; **extremely significant correlation.

**Table 5:** Correlation coefficients between heavy metal concentrations and topographic factors.

	Mn	Ni	As	Cu	Zn	Pb	Cr
DEM	0.050	− 0.044	0.027	− 0.084	0.047	0.062	− 0.064
Aspect	− **0.054**	− **0.180**	− **0.307**	− **0.299**	− **0.111**	0.017	− **0.369**
Slope	0.025	− 0.026	− 0.091	− 0.031	0.071	**0.134**	− 0.101

Significant values are in bold.

*Significant correlation; **extremely significant correlation.

The topographic factors included DEM, aspect and slope, and the correlation between the concentration of each heavy metal element and the topographic factors was calculated separately (Table 5). The coefficients in Table 5 show that the concentrations of six metal elements (Mn, Ni, As, Cu, Zn, and Cr) had the best correlation with aspect, that is they had the greatest effect on the metal elements, and the concentration of Pb had the best correlation with slope. However, the correlation coefficients of each terrain factor with metals were relatively low, and other factors must be considered in the subsequent selection of the modeling factors to select the most appropriate terrain factor.

Sentinel-2A images of the study area were processed using PCA to extract the PC4 component that could reflect the alteration information and analyze the correlation between the concentration of each heavy metal element and the alteration information (Table 6). From the data in Table 7, it can be seen that, overall, the concentrations of five metal elements, Mn, Ni, Cu, Zn, and Cr, were well correlated with iron-stained alteration, and As and Pb had good correlations with –OH and CO_3_^2−^ alteration. However, the correlation coefficients between the concentration of each metal element and the alteration information did not differ significantly from each other.

**Table 6:** Correlation coefficients between heavy metal element concentrations and etching information.

	Mn	Ni	As	Cu	Zn	Pb	Cr
–OH and CO_3_^2−^ alteration	0.022	0.047	**0.048**	0.011	0.050	**0.050**	− 0.007
Iron-stained alteration	− **0.264**	− **0.199**	0.032	− **0.088**	− **0.251**	0.033	**0.010**

Significant values are in bold.

*Significant correlation; **extremely significant correlation.

**Table 7:** Factor screening for modeling heavy metal element concentrations.

Cr	Modeling factors					
	Green	MNDWI	DVI	CRM	Aspect	Iron-stained alteration
As	Green	Blue	MNDWI	MSAVI	Aspect	–OH, CO_3_^2−^ alteration
Cu	Green	EVI	MNDWI	NDVI	Aspect	Iron-stained alteration
Mn	Green	MNDWI	DVI	RVI	Aspect	Iron-stained alteration
Ni	SWIR2	Blue	MNDWI	EVI	Aspect	–OH, CO_3_^2−^ alteration
Pb	SWIR2	EVI	RGRI	RVI	Slope	Iron-stained alteration
Zn	Green	MNDWI	MSAVI	CRM	Aspect	Iron-stained alteration

According to the results of the above correlation analysis, modeling factors with higher correlation coefficients were screened for the complex covariance test by comprehensively considering the spectral information, topographic factors, and mineral alteration information. Factors with VIF values greater than 10 were discarded, and the final modeling factors that were retained are listed in Table 7.

### Inverse modeling of soil heavy metal content

The original data is first randomly divided into training and validation sets in the ratio of 7:3. For the modeling factors selected for each heavy metal element in the study area, the RFR algorithm was used to construct an inverse model of the soil heavy metal concentrations in the region. The inversion results are listed in Table 8. As shown in Table 8, the prediction accuracy of the concentration of each element was improved by adding alteration information. The elemental concentration of Cu was predicted to be the best with an RMSE of 3.309, MAPE of 11.072%, and R^2^ of 0.904. This was followed by As, Ni, and Zn, all with R^2^ greater than 0.5 and significantly lower RMSE and MAPE. Although the prediction of the elemental concentrations of Pb, Cr, and Mn was improved by adding the alteration information, the prediction accuracy remained average. The analysis revealed that the low prediction accuracy of Cr and Pb may be due to the presence of a high number of outliers in the prediction model.

**Table 8:** Comparison of the accuracy of models for predicting soil heavy metal concentrations.

	Spectral information + Terrain factor			Spectral information + Terrain factor + Alteration information		
	RMSE	MAPE (%)	*R*^2^	RMSE	MAPE (%)	*R*^2^
Cu	3.473	15.296	0.439	3.309	11.072	**0.904**
Zn	6.147	10.432	0.161	5.065	8.010	**0.523**
Pb	10.190	32.648	0.041	9.241	17.630	0.312
Cr	13.428	15.039	0.055	17.491	15.327	0.394
Mn	43.348	9.401	0.254	36.149	8.201	0.385
As	2.832	15.514	0.270	1.180	13.134	**0.674**
Ni	1.751	7.053	0.290	1.451	5.811	**0.545**

Significant values are in bold.

**Figure 4:**
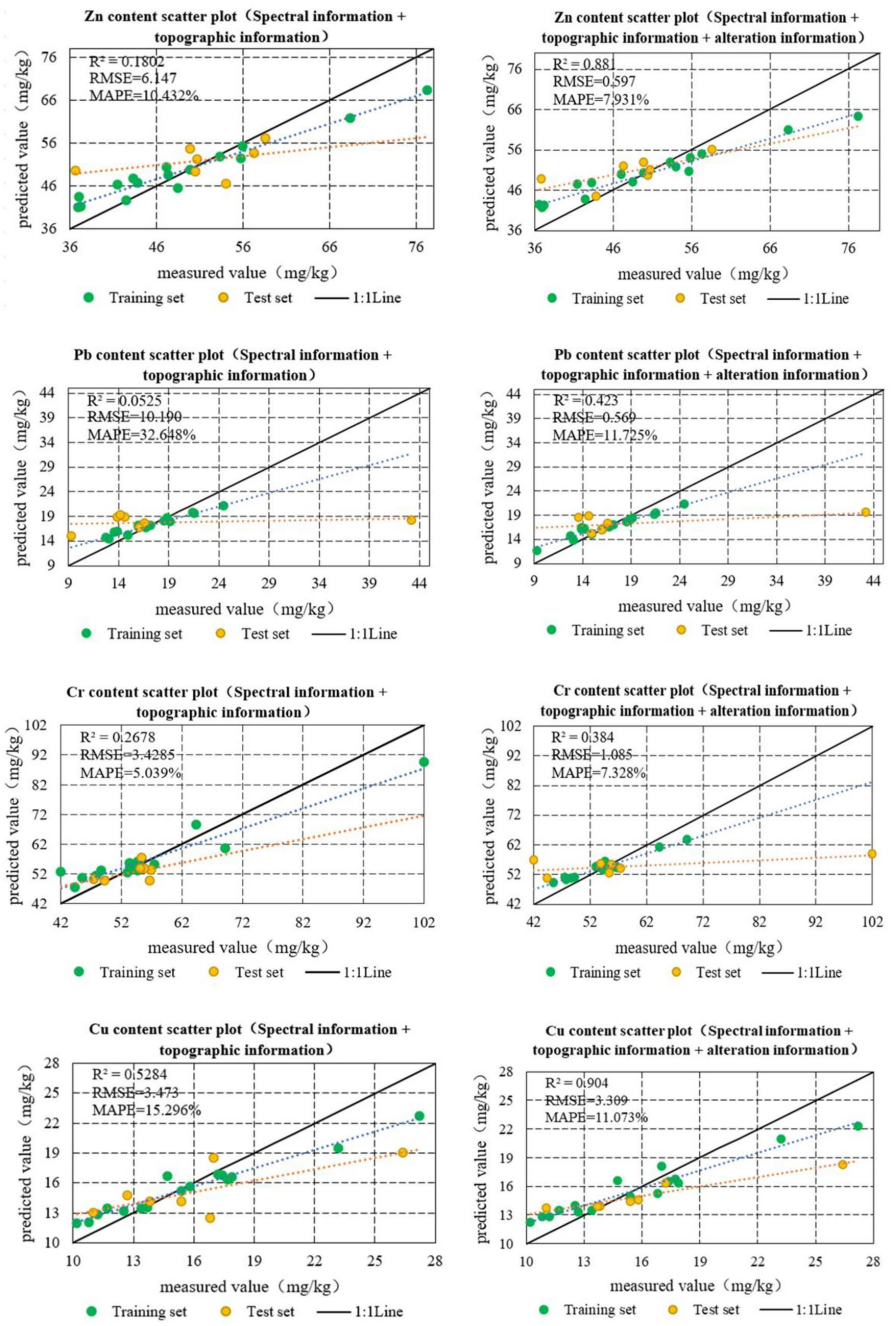

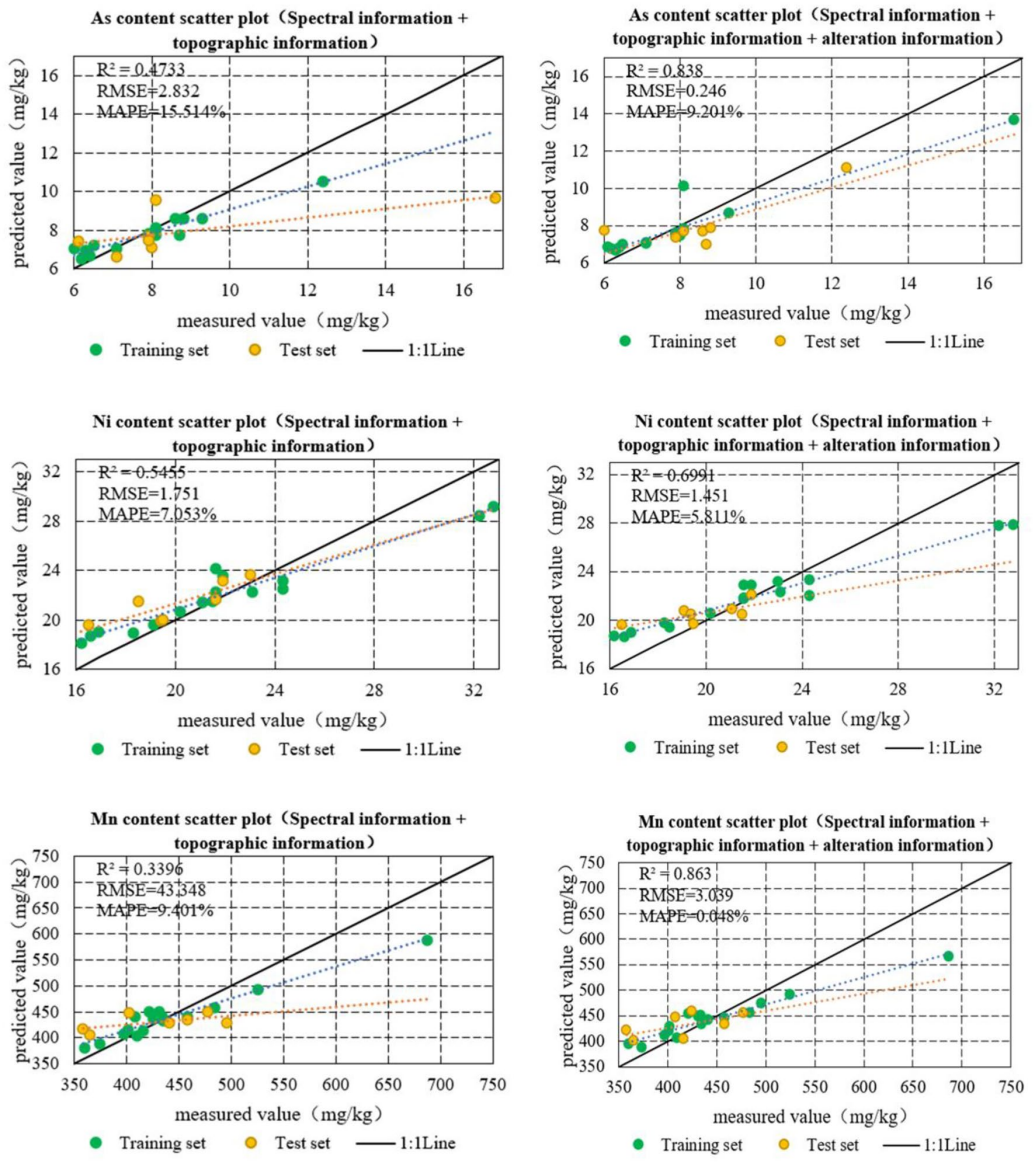
Comparison of the accuracy of inversion models for heavy metal concentrations.

The corresponding scatter plots were obtained by comparing the measured values with the predicted concentrations of each heavy metal in the study area (Fig. 4). Statistical analysis of the predicted and measured values revealed that the inclusion of alteration information improved the accuracy of the model, and the prediction results were more stable. The scatterplot of the total sample shows that the closer it is to the y = x line, the better the model fit. The sample-predicted and measured values of the RFR model with the addition of alteration information were mostly concentrated around the 1:1 line, and the model fitting was superior to that predicted by the model without the addition of alteration information.

### Spatial distribution of soil heavy metal content

**Figure 5:**
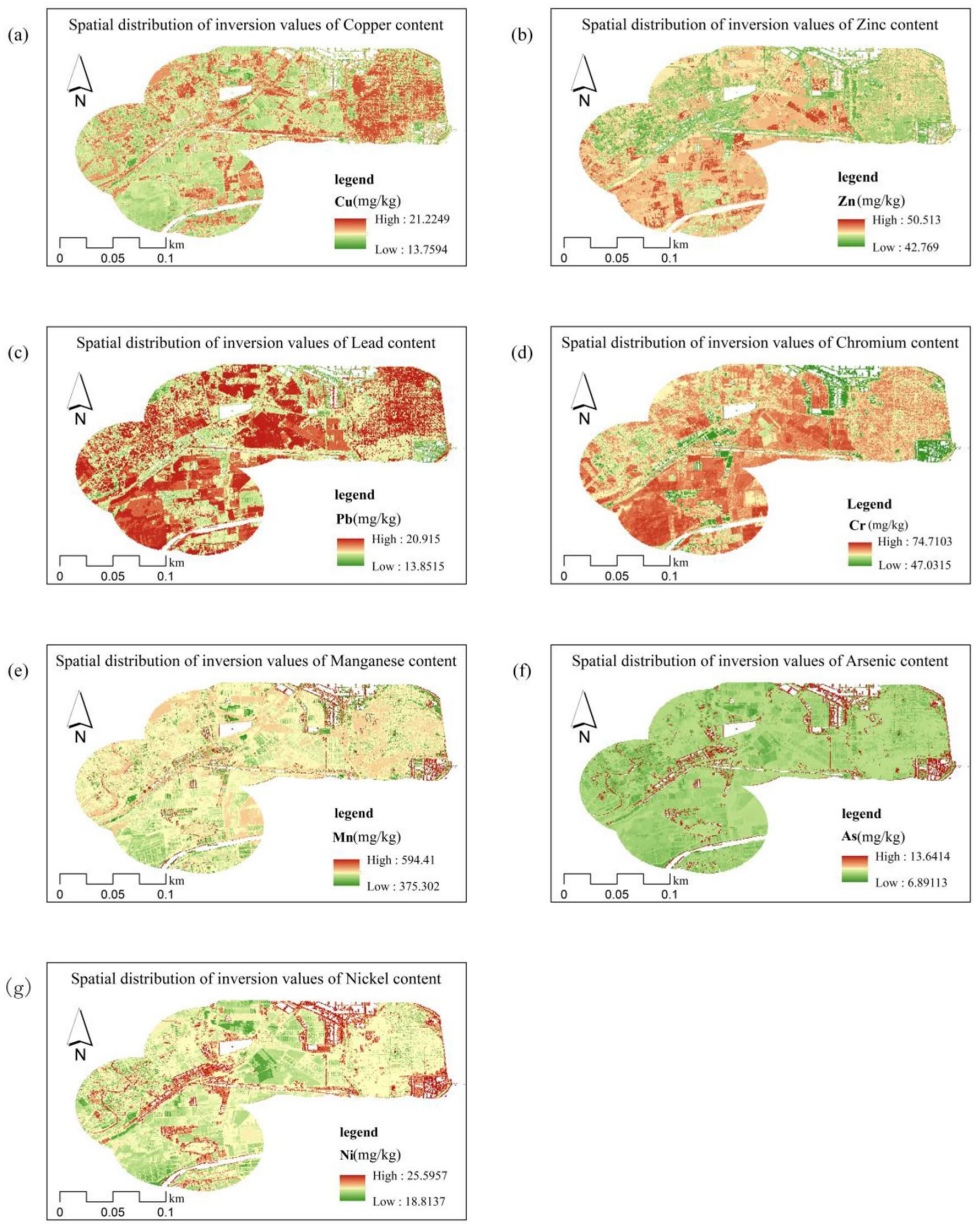
Spatial distribution of heavy metal element concentrations from Random Forest Modeling.

The spatial distribution of heavy metal concentrations was estimated using the constructed RF model based on spectral derivative indices, topographic factors, and alteration information after screening for the seven heavy metal elements in the study area. The inversion results are shown in Fig. 5. The analysis results showed that the spatial distribution of the elemental concentrations of As, Ni, and Mn was generally consistent, with the high-concentration areas mainly distributed along the banks of rivers, lakes, and pits, followed by higher concentrations in the eastern oil extraction areas and lower concentrations in the agricultural areas. The Yellow River carries a large amount of sediment downstream from its source, and some of the sediments form piles along the banks. High concentrations on both sides of the river and around the lake may be due to compound industrial and agricultural pollution upstream, which causes heavy metals to accumulate. As a whole in the study area, heavy metal Cu concentrations in soils are low in the northwestern and central regions. The high-concentration area of Cu is mainly concentrated in the eastern oil extraction area, both sides of the river, and around the lake. Among them, the distribution of Cu in the soil of the eastern oil extraction area is continuous and the concentration is significantly higher than that in other areas. It is presumed that the Cu pollution in the area is related to the oil and gas development of the oil extraction plant. The areas with high concentrations of Zn were mainly distributed in the farmland areas on both sides of the river, with an overall trend of high in the southwest and low in the northeast. A combination of images and field surveys revealed that the southwestern side of the study area is mostly dominated by agricultural fields, suggesting that the high concentrations of Zn in the soil are likely to come from agricultural production practices. Areas with high concentrations of Cr and Pb were more widely distributed, with lower concentrations only in some of the pits and ponds. Both have a continuous distribution of heavy metal content in the soils of the area, with higher contamination on both sides and in the center. The spatial distributions of the concentrations of the seven elements matched well with the results of the correlation analysis. The distribution of the Cr elemental concentration is quite different from that of As, Ni, Mn, and Cu, which is attributed to the low accuracy of the prediction model for the Cr elemental concentration.

Except Cr element, the spatial distribution maps of the concentration of the remaining six heavy metal elements based on spectrally derived indices, topographic factors and alteration information using RF model are similar to the spatial distribution maps of the metals obtained by Yu et al.^41^. using the kriging interpolation method. The feasibility of using mineral alteration information extracted based on remote sensing images for predicting heavy metal content is again demonstrated. Moreover, the spatial distribution maps of heavy metal element concentrations obtained based on this paper's method are more detailed than those obtained based on the Kriging interpolation method. In terms of the drivers of heavy metal aggregation, the causes of Cu, Pb, and Zn aggregation analyzed in this paper have similarities with the sources of Cu, Pb, and Zn aggregation analyzed in the study by Zhang et al.^42^. It provides a more scientific basis for further monitoring the heavy metal content of soil on a large scale and strengthening regional land quality management.

## Conclusions

This study used the town of Gudao in the Yellow River Delta as the study area and obtained seven heavy metal concentrations from 23 sampling points through on-site sampling and analysis. Based on Sentinel-2A images, spectral derivative indices, terrain factors, and mineral alteration information were extracted and filtered. A soil heavy metal concentration inversion model was constructed using the RFR algorithm, and the accuracy of the model was evaluated to achieve the spatial distribution inversion of heavy metal concentrations within the region. The results of this study are as follows:

–OH and CO_3_^2−^ alterations can be effectively used for the inverse modeling of soil As and Ni contents. The RMSE and MAPE metrics of the prediction model were significantly reduced, and the R^2^ improved from 0.2 to more than 0.5. Improvement of prediction by applying iron-stained alterations to Cr, Cu, Pb, Zn, and Mn elemental concentration inversion. In particular, the prediction accuracy of Cu elemental concentration was significantly improved with an RMSE of 3.309, MAPE of 11.072%, and R2 of 0.904, followed by elemental Zn with an RMSE of 5.065, MAPE of 8.01%, and R2 of 0.523, whereas the accuracy of Mn, Cr, and Pb improved and remained at a low level. However, for the entire sample, the correlation between the measured and predicted values of Mn was better, and the low prediction accuracy of Cr and Pb may have been due to the existence of points with higher concentrations.
2. The spatial distribution of soil heavy metal concentrations in the study area was inverted based on the constructed model. It was found that the areas of high concentration of As, Ni and Mn elements were mainly located on the banks of rivers, around lakes and ponds, followed by higher concentrations in the eastern oil extraction areas and lower concentrations in the agricultural areas. The high-concentration area of Cu is consistent with the distribution of the high-concentration areas of the above three elements, mainly in the eastern oil extraction area, both sides of the river, and around the lake. Areas with high Zn concentrations were mainly located in agricultural areas on both sides of the river. High concentrations of Cr and Pb are distributed over a wide area, with lower concentrations found only in some areas of the pits. The results of this study provide a scientific basis for the assessment and management of soil quality. For the perimeters of points with abnormally high heavy metal concentrations, further research is required in terms of field investigation, data analysis, and model construction. The number of sampling points can be increased and the range can be further expanded so that more data can be used for modeling and the applicability of the model can be improved. Meanwhile, at the level of spectral response mechanism, the correlation study between heavy metal elements and alteration information needs to be further deepened.

## Data Availability

The datasets generated during the current study are not publicly available due the procedures established in contract with the funding institution but are available from the corresponding author on reasonable request.
